# Noninvasive Blood Glucose Concentration Measurement Based on Conservation of Energy Metabolism and Machine Learning

**DOI:** 10.3390/s21216989

**Published:** 2021-10-21

**Authors:** Jianming Zhu, Yu Zhou, Junxiang Huang, Aojie Zhou, Zhencheng Chen

**Affiliations:** 1 School of Life and Environmental Sciences, Guilin University of Electronic Technology, Guilin 541004, China; zhujianming@guet.edu.cn (J.Z.); zhouyu5859@163.com (Y.Z.); 19122202015@mails.guet.edu.cn (A.Z.); 2 School of Electronic Engineering and Automation, Guilin University of Electronic Technology, Guilin 541004, China; 19082304011@mails.guet.edu.cn

**Keywords:** multisensor fusion, diabetes, metabolic heat production, regression model, noninvasive glucose concentration detection, wrist

## Abstract

Blood glucose (BG) concentration monitoring is essential for controlling complications arising from diabetes, as well as digital management of the disease. At present, finger-prick glucometers are widely used to measure BG concentrations. In consideration of the challenges of invasive BG concentration measurements involving pain, risk of infection, expense, and inconvenience, we propose a noninvasive BG concentration detection method based on the conservation of energy metabolism. In this study, a multisensor integrated detection probe was designed and manufactured by 3D-printing technology to be worn on the wrist. Two machine-learning algorithms were also applied to establish the regression model for predicting BG concentrations. The results showed that the back-propagation neural network model produced better performance than the multivariate polynomial regression model, with a mean absolute relative difference and correlation coefficient of 5.453% and 0.936, respectively. Here, about 98.413% of the predicted values were within zone A of the Clarke error grid. The above results proved the potential of our method and device for noninvasive glucose concentration detection from the human wrist.

## 1. Introduction

As a lifelong metabolic disorder, diabetes mellitus, or diabetes, not only poses serious threats to human life and health, but also places an economic burden on society [1,2]. The number of people diagnosed with diabetes is increasing rapidly every year. According to the reports of the International Diabetes Federation, as of 2019, the global number of patients with diabetes had increased from 151 million to 463 million over 20 years [3]. Almost half of the possible candidates for diabetes are not yet clinically diagnosed. In addition, diabetes is predicted to affect an additional 237 million people worldwide over the next 26 years [3]. Thus, diabetes is recognized as one of the global top-10 chronic diseases of the 21st century. Long-term diabetes can lead to changes in the dental, cardiovascular, and renal systems, as well as lower limbs and other tissues and organs [4]; these complications may cause pain in the diabetic population. Blood glucose (BG) concentration monitoring is one of the main methods of controlling diabetes [5]. At present, the most commonly used method for BG detection is via finger-prick testing [6], in which the BG concentration is measured by collecting blood from the capillaries in the fingertip and placing it on an enzymatic test strip attached to a glucometer. However, this technique carries the risk of wound infection and loss of fingertip sensitivity over time. Owing to the negative impacts of long-term invasive BG detection on the quality of life, the diabetic population is unable to monitor BG concentrations as required by doctors. Thus, noninvasive BG measurements have been recognized as ideal means to manage BG levels in patients with diabetes.

Over the past few years, noninvasive BG detection techniques have developed rapidly, with innovations concerning the principle of BG detection, measurement site, and detection equipment. Yu [7], Sim [8], Li [9], Tanaka [10], Burmeister [11], and others proposed a noninvasive BG measurement method using near-infrared spectroscopy, which has been applied to various measurement sites such as the finger, earlobe, tongue, nasal septum, and lip. However, this technique is not only affected by the contact pressure and temperature, but also is prone to interference from humidity, skin hydration, and atmospheric pressure. Tran [12] and Mohammadifar [13] et al. proposed the electrochemical and optical monitoring of BG concentration in urine. The drawbacks of this method included the inability to track BG concentrations and its inability to be used for clinical measurements. Gao [14], Park [15], Singh [16], and others proposed extracting the BG values from biofluids, such as sweat, tears, and saliva. However, the relationship between glucose levels in biofluids and that in the blood needs further investigation, and there is a lag in the glucose values measured using such techniques. In recent years, big data and artificial intelligence have developed rapidly and have been applied in the field of biomedicine. Monte-Morene [17], Habbu [18], Zhang [19] and others extracted large numbers of time- and frequency-domain characteristic parameters from photoplethysmography (PPG), and used machine-learning and deep-learning algorithms to predict BG concentrations with good accuracy. However, this method can only be used to classify BG levels, and the physiological relationships between the characteristic parameters and BG concentration are still unknown. Cho et al. [20] proposed noninvasive BG concentration measurements using metabolic heat conformation; however, this method is imperfect. On the one hand, the evaporative heat dissipation is ignored, which seriously affects the metabolic heat production calculations; on the other hand, the pulse rate derived from arterial flow, which is equal to the heart rate (HR), is neglected, and the pulse rate changes directly affect the blood supply to the body, which will inevitably affect the blood concentration and metabolic heat production in the human body.

This paper presents the theory behind BG concentration detection by the metabolic heat conformation method, and establishes a mathematical model for BG concentration detection based on the conservation of energy metabolism. In addition, a method for calculating the metabolic heat production of the body is proposed, and a multisensor integrated detection probe worn on the wrist was designed to facilitate acquisition of multiple physiological parameters. Two machine-learning algorithms, multivariate polynomial regression, and back-propagation neural network (BPNN), were used to establish the regression model to predict BG concentrations in this work. Experiments were performed to demonstrate the feasibility of noninvasive BG concentration detection based on conservation of energy metabolism.

## 2. Principles and Methods

### 2.1. Conservation of Energy Metabolism

**Figure 1:**
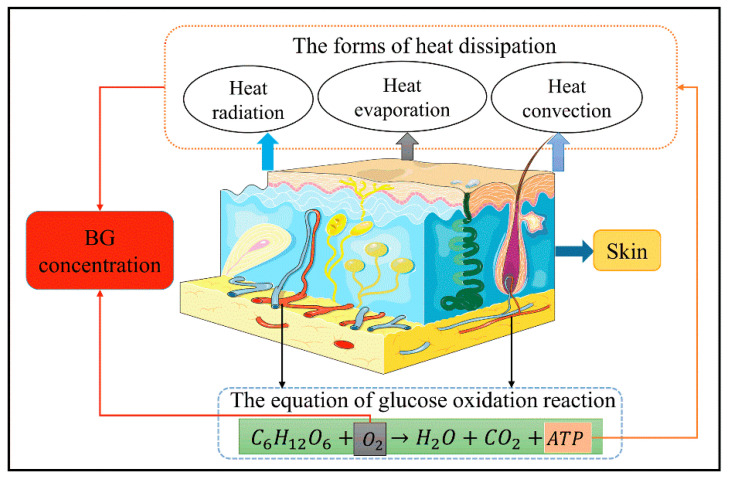
Schematic representation of the human metabolism.

Most of the energy required for human activities in daily life are derived from sugars [21]. The BG in the body is involved in oxidation reactions to produce adenosine triphosphate (ATP); when sufficient oxygen is available, the BG is linearly related to the energy produced by metabolism [22,23]. In addition to the external work done by the body, the energy is specifically converted to heat energy, which is transmitted outwards in different forms, such as heat radiation, evaporation, and convection [24]. The overall process of human metabolism is shown in Figure 1. As the main source of energy, changes in the BG concentration will inevitably cause changes in the human metabolism, which will affect the changes to the physiological parameters, such as body temperature. Hence, BG concentrations can be indirectly detected by measuring the relevant physiological parameters of the human body and establishing a related mathematical model. However, it is important to note that the body must remain at rest when using this method to measure BG concentrations.

We modified and optimized the metabolic heat conformation technique to establish the law of conservation of energy metabolism using the following assumptions:

(a) The human body is a thermal balance system, and the amount of heat generated is equal to the amount of heat dissipated.
(b) The main modes of heat dissipation from the body are thermal radiation, thermal convection, and thermal evaporation.
(c) The external work done by the human body at rest is 0.
(d) The oxygen content in human blood is related to the HR and blood flow rate (BF).
(e) The metabolic heat production of the human body is related to the BG concentration, oxygen saturation (SpO_2_), and HR.

Based on the above assumptions, we proposed a mathematical model for noninvasive BG detection based on the conservation of energy metabolism, as shown in Equation (1):

(1) $$BG=F\left(H,HR,SpO_{2},BF\right)$$

where *F* represents a nonlinear function. The variables in this model are metabolic heat production (*H*), *HR*, *SpO*_2_, and *BF*. All these variables can be measured from the human wrist.

### 2.2. Calculation of Metabolic Heat Production

We proposed a new approach to calculate heat production from metabolism. When the human body is at rest, according to the law of conservation of energy metabolism:

(2) H=M

(3) M=R+C+E

where *M* represents the metabolic heat dissipation, *R* represents the heat transferred through radiation, *C* represents the heat dissipated through convection, and *E* represents the quantity of heat lost through evaporation. *R* can be obtained according to the modified Planck’s law, as shown in Equation (4):

(4) $$R=\varepsilon \sigma T_{r}^{4}$$

where *ε* represents the black body coefficient, which is equal to 1. The relative radiation capacity of the human skin is close to the black body coefficient, so *ε* was set to an empirical value of 0.96 in this work. *σ* is the Stephen–Boltzmann constant, equal to 5.67 × 10^−8^; and *T_r_* is the absolute radiation temperature value of the wrist.

According to Newton’s law of cooling, *C* can be obtained from Equations (5) and (6):

(5) $$C=h_{c}\left(t_{w}-t_{s}\right)$$

(6) $$h_{c}=2.38{\left(t_{w}-t_{s}\right)}^{0.25}$$

where $h_{c}$ is the convective heat transfer coefficient of the wrist surface, $t_{w}$ is the temperature of the human wrist, and $t_{s}$ is the ambient temperature around the body.

Water transforms from a liquid to vapor on the surface of the skin and in mucous membranes, which consumes a large quantity of heat. Thus, *E* can be calculated from the following formulas:

(7) $$E=rm\left(P_{sk}^{*}-P_{a}\right)$$

(8) $$P_{sk}^{*}=0.256t_{w}-3.37$$

(9) $$P_{a}=H_{e}\times P_{sk}^{*}$$

where *r* is the latent heat of vaporization of water, *m* is the permeability coefficient of the skin on the wrist,$\;P_{sk}^{*}$ is the saturation and partial pressure of water vapor in air at the wrist-skin temperature, $P_{a}$ is the partial pressure of water vapor in air at the ambient temperature and humidity, and $H_{e}$ is the environmental humidity around the body.

According to Equations (2)–(9), the equation for calculating *H* is as follows:

(10) $$H=\varepsilon \sigma T_{r}^{4}+2.38{\left(t_{w}-t_{s}\right)}^{1.25}+3.054\left(0.256t_{w}-3.37\right)\left(1-H_{e}\right)$$

where the parameters in Equation (10) are the same as those in Equations (2)–(9).

### 2.3. Calculation of SpO_2_ and HR

Blood oxygen saturation is generally measured by the optical method [25], and the difference in the absorption of near-infrared light of a specific wavelength band (usually between 660 and 940 nm) by the oxyhemoglobin and deoxyhemoglobin in blood is used for detection. Since the wrist contains bones and other dense tissues, the commonly used transmissive PPG sensor is not applicable, and only the reflective PPG sensor can be used. According to the modified Lambert Beer’s law, *SpO*_2_ can be calculated as follows:

(11) $$SpO_{2}=\frac{C_{HbO_{2}}}{C_{HbO_{2}}+C_{Hb}}\times 100\%$$

where $C_{HbO_{2}}$ and $C_{Hb}$ respectively represent the concentrations of oxygenated and deoxygenated hemoglobin in the blood. Then:

(12) $$r=\frac{K_{1}^{\lambda_{red}}C_{HbO_{2}}+K_{2}^{\lambda_{red}}C_{Hb}}{K_{1}^{\lambda_{ir}}C_{HbO_{2}}+K_{2}^{\lambda_{ir}}C_{Hb}}$$

where $K_{1}^{\lambda_{red}}$ and $K_{1}^{\lambda_{ir}}$ are the absorption coefficients of oxyhemoglobin; $K_{1}^{\lambda_{red}}$ and $K_{1}^{\lambda_{ir}}$ are the absorption coefficients of deoxyhemoglobin under red light and near-infrared light, respectively; and r is the light intensity absorption ratio of the wrist for two different wavelengths (660 and 880 nm).

Combining Equations (11) and (12), the$\;SpO_{2}$ can be calculated as follows:

(13) $$SpO_{2}=\frac{K_{2}^{\lambda_{ir}}r-K_{2}^{\lambda_{red}}}{\left(K_{2}^{\lambda_{ir}}-K_{1}^{\lambda_{ir}}\right)r+\left(K_{1}^{\lambda_{red}}-K_{2}^{\lambda_{red}}\right)}\times 100\%$$

According to Equation (13), as long as *r* can be calculated, the value of blood oxygen saturation can be acquired. The calculation of *HR* is as shown in Equation (14):

(14) $$HR=\frac{F_{s}\left(N_{peak}-1\right)}{x_{end}-x_{start}}\times 60$$

where $F_{s}$ the sampling frequency, $N_{peak}$ is the number of peaks of the PPG signal in a given period of time, and $x_{start}$ and $x_{end}$ are the coordinate positions of the first and last wave crests, respectively.

## 3. System Composition

**Figure 2:**
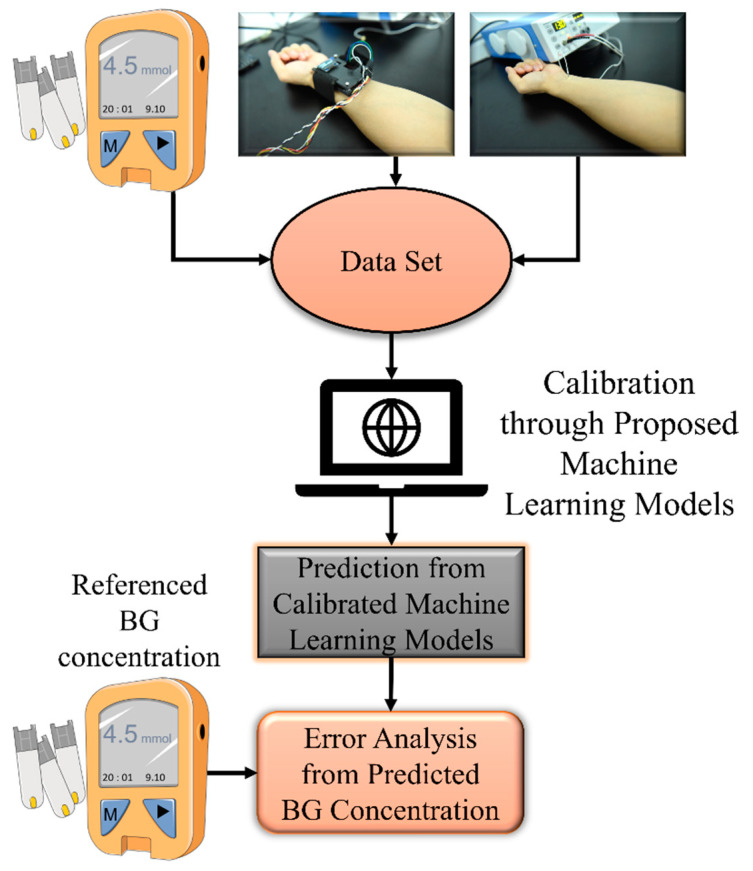
Operation process of the proposed device.

The proposed measurement system was mainly composed of three parts, namely the multisensor integrated detection probe, a laser Doppler blood-flow meter, and a computer for establishing the noninvasive BG detection algorithm. The working process of the system is shown in Figure 2.

### Design of Multisensor Integrated Detection Probe

**Figure 3:**
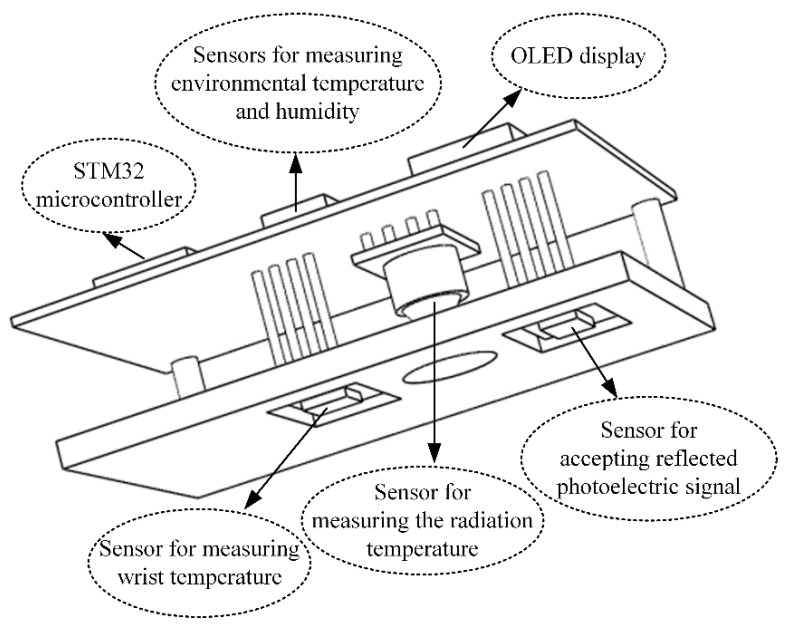
The 3D structure of the multisensor integrated detection probe.

Herein, a multisensor integrated detection probe based on the conservation of energy metabolism was designed to measure multiple physiological parameters. The probe included a thermal radiation sensor, two temperature and humidity sensors, and a reflective PPG sensor. The reflective PPG sensor could obtain two different PPG signals, which could be decomposed into those from 660 nm red light and 880 nm near-infrared light. From the sensing principle described above, $T_{r}$, $t_{w}$, $t_{s}$, $H_{e}$, and PPG could be measured; further, H, SpO_2_, and HR could be calculated using these parameters. The detailed calculation methods are as noted in Section 2. The radiation sensor needed to be placed at a certain distance from the wrist surface, and the temperature sensor had to be in contact with the skin surface. The reflective PPG sensor was enclosed in a light-avoiding sponge to reduce interference from ambient light. The size of the detection probe was 70 mm × 40 mm × 18 mm. The 3D structure of the probe is shown in Figure 3.

**Figure 4:**
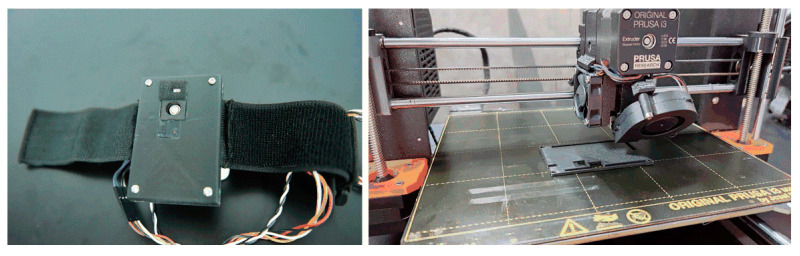
Physical views of the multisensor detection probe and 3D-printed device.

Figure 4 shows the physical views of the multisensor integrated detection probe and its 3D-printed equivalent device.

## 4. Machine-Learning Models for BG Level Prediction

### 4.1. Multiple Polynomial Regression

A multiple polynomial regression of the fourth degree (MPR4) was used to predict the BG concentration. To reduce collinearity, only the interaction terms were generated. The kernel function of the polynomial regression is as follows:

(15) $$\begin{array}{ll} y\;=\;a_{0}\;+\;a_{1}x_{1} & +\;a_{2}x_{3}\;+\;a_{3}x_{3}\;+\;a_{4}x_{4}\;+\;a_{5}x_{1}x_{2}\;+\;a_{6}x_{1}x_{3}\;+\;a_{7}x_{1}x_{4}\\ & +\;a_{8}x_{2}x_{3}\;+\;a_{9}x_{2}x_{4}\;+\;a_{10}x_{3}x_{4}\;+\;a_{11}x_{1}x_{2}x_{3}\\ & +\;a_{12}x_{1}x_{2}x_{4}\;+\;a_{13}x_{1}x_{3}x_{4}\;+\;a_{14}x_{2}x_{3}x_{4}\\ & +\;a_{15}x_{1}x_{2}x_{3}x_{4} \end{array}$$

**Figure 5:**
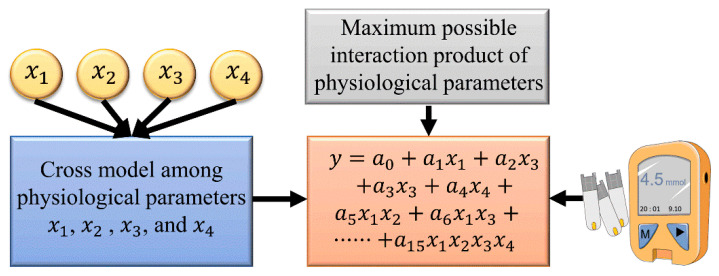
The MPR4 model used for prediction.

In the above expression, H, SpO_2_, HR, and BF were defined as $x_{1}$,$\;x_{2}$,$\;x_{3}$, and $x_{4}$ predictors, respectively, whereas y was the predicted BG concentration. The overall diagram of the MPR4 model is presented in Figure 5. Here, $a_{1}–a_{15}$ were the regression coefficients, and $a_{0}$ was a compensation parameter. The proposed MPR4 was a multivariate nonlinear regression model, and a total of 15 customized interaction variables were defined based on the predictors. The MPR4 model is justifiable when the sample size is greater than three times the number of variables.

### 4.2. BPNN

Artificial neural networks are effective regression models that not only fit linear relationships, but also have good ability to describe nonlinear relationships [26,27]. The BPNN is a type of feedforward neural network that is widely applied to modeling problems in engineering. Thus, the BPNN was examined here for BG level prediction. The network consisted of three layers; namely, the input, output, and hidden layers [28]. Tansig sigmoid functions were used for the hidden layer, and the Levenberg–Marquardt back-propagation algorithm was used to train the model. For artificial neural networks with only three layers, Kolmogorov provided the equivalence between the numbers of neurons in the input and hidden layers [29], as follows:

(16) $$N_{hid}=2N_{in}+1$$

**Figure 6:**
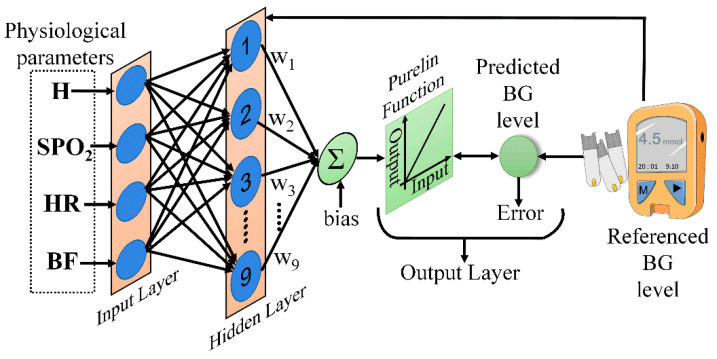
The BPNN model used for prediction.

The overall representation of BPNN model is shown in Figure 6.

### 4.3. Model Evaluation Indexes

The mean absolute relative difference (*mARD*), correlation coefficient (*CORR*), mean absolute deviation (*MAD*), and root mean square error (*RMSE*) were calculated to evaluate the performance of MPR4 and BPNN models. The formulas for computing the above estimation indexes are as follows:

(17) $$mARD=\frac{1}{n}\sum_{i=1}^{n}\frac{\left|Y_{i}-X_{i}\right|}{X_{i}}\times 100\%$$

(18) $$CORR=\frac{\sum_{i=1}^{n}\left(X_{i}-{\bar{X}}_{i}\right)\left(Y_{i}-{\bar{Y}}_{i}\right)}{\sqrt{\sum_{1}^{n}{\left(X_{i}-{\bar{X}}_{i}\right)}^{2}{\left(Y_{i}-{\bar{Y}}_{i}\right)}^{2}}}$$

(19) $$MAD=\frac{1}{n}\overset{n}{\underset{i=1}{\sum }}\left|Y_{i}-{\bar{Y}}_{i}\right|$$

(20) $$RMSE=\sqrt{\frac{\sum_{i=1}^{n}{\left|\left(X_{i}-Y_{i}\right)\right|}^{2}}{n}}$$

where $X_{i}$ and $Y_{i}$ are the reference BG value and the predicted BG value, respectively; ${\bar{X}}_{i}$ and ${\bar{Y}}_{i}$ are the means of measured and predicted BG concentrations, respectively; and *n* is the number of samples.

## 5. Experiments and Results

### 5.1. Experimental

H, SpO_2_, and HR values were determined using the developed multisensor detection probe. The extraction equipment for BF was obtained from a laser Doppler blood-flow meter (type: PeriFlux System 5000), and the test equipment used to measure the reference BG concentration was a finger-prick glucometer (type: Accu-Chek^®^ Performa) along with its electrochemical test strips.

The experimental procedure was as follows. Prior to obtaining the experimental data, each volunteer was asked to clean their hands with warm water and soap to avoid contamination. The site to be measured was then disinfected with 75% alcohol solution and allowed to dry. At the same time, the multisensor detection probe and laser Doppler blood-flow meter were switched on, and the glucometer test strips were set up. Every volunteer was instructed to sit comfortably in a chair and wait for steady breathing while wearing the multisensor integrated detection probe on their wrist. During data collection, the volunteers remained quiet and maintained a fist with their left hand. To reduce individual variability, the measurement position and contact pressure were kept as constant as possible. In addition, the entire experiment was conducted in an environment with comfortable temperature and humidity. Subsequently, multiple physiological parameters were acquired with the multisensor detection probe, and the corresponding BG concentration and BF were obtained with the finger-prick glucometer and laser Doppler blood-flow meter.

In this study, 12 healthy volunteers were recruited for the experiments. The volunteers were notified of the detailed experimental procedures and potential risks. The experiments were conducted after the volunteers signed informed consent forms, and complied with the ethical standards. The experimental time points were 30 min before and 30 min, 60 min, 90 min, and 120 min after meals. The entire experiment was conducted at three time periods: breakfast, lunch, and dinner. The amount of data acquired and end of data measurement varied depending on the individual conditions of each volunteer; some individuals were involved in only one day of data collection. Ultimately, a total of 211 samples were collected. The maximum and minimum values of the reference BG concentrations were 10 mmol/L and 4.1 mmol/L, respectively.

### 5.2. Performance Comparisons of the Machine-Learning Models

The 211 samples were divided into two groups by the hold-out method, and 118 samples were randomly selected as the training set for model calibration, while the remaining 63 samples were used as the test set for model verification and performance evaluation. The statistical analysis values of the proposed models are shown in Table 1. Compared with the proposed MPR4 model, the proposed BPNN model had a better prediction performance.

### 5.3. Results

**Figure 7:**
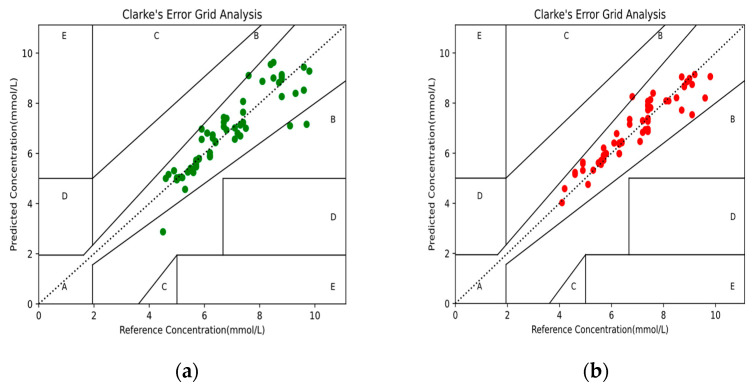
The Clarke error grid analysis of the predicted BG values: (**a**) BG values based on the MPR4 model; (**b**) BG values based on the BPNN model.

The Clarke error grid analysis is considered the clinical gold standard for evaluating the accuracy of measured BG concentration, and was applied to assess the agreement between the predicted and reference BG values [30]. The Clarke error grid analysis was divided into five areas, in which the predicted BG values within area A indicated a deviation of no more than 20% from the reference BG values; values in area B indicated those that were clinically acceptable; and values in areas C, D, and E indicated possible results of inappropriate processing. The Clarke error grid analysis was performed on the predicted BG concentration results from the MPR4 and BPNN models, as shown in Figure 7 and Table 2. In addition, the standard errors of the predicted (SEP) BG values based on the two models are shown in Table 2. The above results showed that the BPNN model had a better predictive performance than the MPR4 model.

## 6. Conclusions

This paper described the feasibility of noninvasive acquisition of BG concentration from the human wrist based on conservation of energy metabolism. In addition, a method to calculate the metabolic heat production in the human wrist was proposed. To facilitate measurement of multiple physiological parameters from the human body, a multisensor integrated detection probe was designed to be worn on the wrist. An efficient regression model was established to measure BG concentration, and a group of 12 healthy volunteers was recruited for the clinical trial. It was calculated that mARD and SEP were 5.453% and 0.159 mmol/L, respectively. The CORR between the referenced and predicted BG values was 0.936. Furthermore, about 98.413% of the predicted BG values were within area A of the Clarke error grid. The proposed device and method have great potential for monitoring BG concentration from the human wrist.

In the future, additional physiological data must be collected from the diabetic population to expand the detection range of BG concentrations using the proposed method, and serum glucose should be used as the reference value to improve the accuracy of the measurement. In addition, a device for detecting blood-flow velocity could be integrated with the probe to realize a wearable noninvasive glucometer for real-time monitoring of human BG concentrations.

## Figures and Tables

**Table 1:** The performance of the two models for statistical analysis.

Model	mARD (%)	CORR	MAD (mmol/L)
MPR4	6.703	0.893	1.198
BPNN	5.453	0.936	1.084

**Table 2:** Performance comparison of the MPR4 and BPNN models.

Regression Model	RMSE mmol/L	SEP mmol/L	Clarke Error Grid Analysis (%)				
			A	B	C	D	E
MPR4	0.676	0.186	95.238	4.762	0.000	0.000	0.000
BPNN	0.505	0.159	98.413	1.587	0.000	0.000	0.000

## Data Availability

Not applicable.

## References

[1] Inzucchi S.E., Bergenstal R.M., Buse J.B., Diamant M., Ferrannini E., Nauck M., Peters A.L., Tsapas A., Wender R., Matthews D.R. (2015). Management of Hyperglycemia in Type 2 Diabetes, 2015: A Patient-Centered Approach Update to a Position Statement of the American Diabetes Association and the European Association for the Study of Diabetes. Diabetes Care.

[2] Williams R., Karuranga S., Malanda B., Saeedi P., Basit A., Besançon S., Bommer C., Esteghamati A., Ogurtsova K., Zhang P. (2020). Global and regional estimates and projections of diabetes-related health expenditure: Results from the International Diabetes Federation Diabetes Atlas, 9th edition. Diabetes Res. Clin. Pract..

[3] Saeedi P., Petersohn I., Salpea P., Malanda B., Karuranga S., Unwin N., Colagiuri S., Guariguata L., Motala A.A., Ogurtsova K. (2019). Global and regional diabetes prevalence estimates for 2019 and projections for 2030 and 2045: Results from the International Diabetes Federation Diabetes Atlas, 9th edition. Diabetes Res. Clin. Pract..

[4] Bolla A.S., Priefer R. (2020). Blood glucose monitoring- an overview of current and future non-invasive devices. Diabetes Metab. Syndr. Clin. Res. Rev..

[5] Lee I., Probst D., Klonoff D., Sode K. (2021). Continuous glucose monitoring systems-Current status and future perspectives of the flagship technologies in biosensor research. Biosens. Bioelectron..

[6] Lekha S., Suchetha M. (2020). Recent Advancements and Future Prospects on E-Nose Sensors Technology and Machine Learning Approaches for Non-Invasive Diabetes Diagnosis: A Review. IEEE Rev. Biomed. Eng..

[7] Yu Y., Huang J.-P., Zhu J., Liang S.-L. (2021). An Accurate Noninvasive Blood Glucose Measurement System Using Portable Near-Infrared Spectrometer and Transfer Learning Framework. IEEE Sens. J..

[8] Sim J.Y., Ahn C.-G., Jeong E.-J., Kim B.K. (2018). In vivo Microscopic Photoacoustic Spectroscopy for Non-Invasive Glucose Monitoring Invulnerable to Skin Secretion Products. Sci. Rep..

[9] Li Z., Li G., Yan W.-J., Lin L. (2014). Classification of diabetes and measurement of blood glucose concentration noninvasively using near infrared spectroscopy. Infrared Phys. Technol..

[10] Tanaka Y., Tajima T., Seyama M., Waki K. (2020). Differential Continuous Wave Photoacoustic Spectroscopy for Non-Invasive Glucose Monitoring. IEEE Sens. J..

[11] Burmeister J.J., Arnold M.A. (1999). Evaluation of measurement sites for noninvasive blood glucose sensing with near-infrared transmission spectroscopy. Clin. Chem..

[12] Tran H.V., Nguyen T.V., Nguyen N.D., Piro B., Huynh C.D. (2018). A nanocomposite prepared from FeOOH and N-doped carbon nanosheets as a peroxidase mimic, and its application to enzymatic sensing of glucose in human urine. Microchim. Acta.

[13] Mohammadifar M., Tahernia M., Choi S. (2019). An Equipment-Free, Paper-Based Electrochemical Sensor for Visual Monitoring of Glucose Levels in Urine. Slas Technol..

[14] Gao W., Emaminejad S., Nyein H.Y.Y., Challa S., Chen K., Peck A., Fahad H.M., Ota H., Shiraki H., Kiriya D. (2016). Fully integrated wearable sensor arrays for multiplexed in situ perspiration analysis. Nature.

[15] Park J., Kim J., Kim S.-Y., Cheong W.H., Jang J., Park Y.-G., Na K., Kim Y.-T., Heo J.H., Lee C.Y. (2018). Soft, smart contact lenses with integrations of wireless circuits, glucose sensors, and displays. Sci. Adv..

[16] Singh A.K., Jha S.K. (2019). Fabrication and Validation of a Handheld Non-Invasive, Optical Biosensor for Self-Monitoring of Glucose Using Saliva. IEEE Sens. J..

[17] Monte-Moreno E. (2011). Non-invasive estimate of blood glucose and blood pressure from a photoplethysmograph by means of machine learning techniques. Artif. Intell. Med..

[18] Habbu S., Dale M., Ghongade R. (2019). Estimation of blood glucose by non-invasive method using photoplethysmography. Sadhana-Acad. Proc. Eng. Sci..

[19] Zhang G., Mei Z., Zhang Y., Ma X., Lo B., Chen D., Zhang Y. (2020). A Noninvasive Blood Glucose Monitoring System Based on Smartphone PPG Signal Processing and Machine Learning. IEEE Trans. Ind. Inform..

[20] Cho O.K., Kim Y.O., Mitsumaki H., Kuwa K. (2004). Noninvasive measurement of glucose by metabolic heat conformation method. Clin. Chem..

[21] Hofsteenge G.H., Chinapaw M.J., Waal H.A.D.-V.D., Weijs P.J. (2010). Validation of predictive equations for resting energy expenditure in obese adolescents. Am. J. Clin. Nutr..

[22] Chen Z.-C., Jin X.-L., Zhu J.-M., Wang D.-Y., Zhang T.-T. (2009). Non-invasive glucose measuring apparatus based on conservation of energy method. J. Central South Univ. Technol..

[23] Tang F., Wang X., Wang D., Li J. (2008). Non-invasive glucose measurement by use of metabolic heat conformation method. Sensors.

[24] Miyake R., Tanaka S., Ohkawara K., Ishikawa-Takata K., Hikihara Y., Taguri E., Kayashita J., Tabata I. (2011). Validity of Predictive Equations for Basal Metabolic Rate in Japanese Adults. J. Nutr. Sci. Vitaminol..

[25] Faber D.J., Mik E.G., Aalders M., Van Leeuwen T.G. (2005). Toward assessment of blood oxygen saturation by spectroscopic optical coherence tomography. Opt. Lett..

[26] Pastor-Bárcenas O., Olivas E.S., Martín-Guerrero J.D., Camps-Valls G., Carrasco-Rodríguez J., del Valle-Tascón S. (2005). Unbiased sensitivity analysis and pruning techniques in neural networks for surface ozone modelling. Ecol. Model..

[27] Chuedoung M., Sarika W., Lenbury Y. (2009). Dynamical analysis of a nonlinear model for glucose-insulin system incorporating delays and beta-cells compartment. Nonlinear Anal.-Theory Methods Appl..

[28] Prado-Prado F.J., Garcia-Mera X., González-Díaz H. (2010). Multi-target spectral moment QSAR versus ANN for antiparasitic drugs against different parasite species. Bioorg. Med. Chem..

[29] Cheng J., Ji Z., Li M., Dai J. (2019). Study of a noninvasive blood glucose detection model using the near-infrared light based on SA-NARX. Biomed. Signal Process. Control.

[30] Clarke W.L., Cox D., Gonder-Frederick L.A., Carter W., Pohl S.L. (1987). Evaluating clinical accuracy of systems for self-monitoring of blood glucose. Diabetes Care.

